# Plasmon-Enhanced Controlled Drug Release from Ag-PMA Capsules

**DOI:** 10.3390/molecules25092267

**Published:** 2020-05-11

**Authors:** Giulia Neri, Carmelo Corsaro, Enza Fazio

**Affiliations:** 1 Department of Chemical, Biological, Pharmaceutical and Environmental Sciences, University of Messina, Viale F. Stagno D’Alcontres 31, I-98166 Messina, Italy; 2 Department of Mathematical and Computational Sciences, Physics Science and Earth Science, University of Messina, Viale F. Stagno D’Alcontres 31, I-98166 Messina, Italy; ccorsaro@unime.it (C.C.); enfazio@unime.it (E.F.)

**Keywords:** metal-polymeric composite, silica template, drug nanocarrier, light stimuli drug release, surface plasmon resonance

## Abstract

Silver (Ag)-grafted PMA (poly-methacrylic acid, sodium salt) nanocomposite loaded with sorafenib tosylate (SFT), an anticancer drug, showed good capability as a drug carrier allowing on-demand control of the dose, timing and duration of the drug release by laser irradiation stimuli. In this study, the preparation of Ag-PMA capsules loaded with SFT by using sacrificial silica microparticles as templates was reported. A high drug loading (DL%) of ∼13% and encapsulation efficiency (EE%) of about 76% were obtained. The photo-release profiles were regulated via the adjustment of light wavelength and power intensity. A significant improvement of SFT release (14% vs. 21%) by comparing SFT-Ag-PMA capsules with Ag-PMA colloids under the same experimental conditions was observed. Moreover, an increase of drug release by up to 35% was reached by tuning the laser irradiation wavelength near to Ag nanoparticles’ surface plasmon resonance (SPR). These experimental results together with more economical use of the active component suggest the potentiality of SFT-Ag-PMA capsules as a smart drug delivery system.

## 1. Introduction

Over the last few years, a rapid increase in research on mesoporous silica particles (MSPs) as drug carriers for the treatment of several diseases, has been observed [1,2]. Their widespread applications, regarding the loading of both small molecules and macromolecules such as proteins, siRNA and drugs, were possible because of MSPs being a versatile carrier [3] due to the tunability of their size, morphology, chemical composition, pore size and structure [4,5]. Drug release with simultaneous carrier decomposition has been demonstrated in SiO${}_{2}$-drug composite [6]: drug molecules are easily confined and stabilized within the SiO${}_{2}$ mesopores in a non-crystalline form, which should enhance the drug dissolution rate [7,8]. However, the significant in vivo toxicity of MSPs, ranging from 150 nm to 4 μm, by intravenous and intraperitoneal administration [9,10] (caused by a low stability in water media of MSPs loaded with poorly water soluble drugs [11,12]), limited their applications as drug carriers. To overcome these drawbacks, several parameters, such as particle size, morphology, chemical composition and pore size have been investigated [13]. Vallhov et al. reported better viability, uptake and immune regulatory markers for bigger MSPs compared to smaller ones [14]: the sizes of MSPs can influence their biocompatibility and water stability, according with administration pathway. At the same time, a suitable functionalization of the large surface area of MSPs was widely investigated in the medicine field and it turned out to be an important tool to overcome the toxicity issue of these systems [15].

Nanoparticles (NPs) with hydrophobic inner cores and hydrophilic outer shells have received great attention for their superior properties in drug delivery of poorly hydrophilic drugs [16], such as sorafenib. Sorafenib and its tosylate salt (sorafenib tosylate, SFT) have attracted considerable attention due to their ability to inhibit several tyrosine protein kinases which are involved in the tumor progression and angiogenesis. Pre-clinical tests showed the ability of sorafenib to inhibit tumor cell proliferation and vascularization by the activation of the receptor for tyrosine kinase signaling in the *Ras/Raf/Mek/Erk* cascade pathway [17,18]. Thus, sorafenib was approved for the treatment of specific tumors, such as thyroid cancer, primary kidney cancer and advanced primary liver cancer. Unfortunately, its clinical applicability is limited by poor aqueous solubility (LogP = 3.8) and undesirable side effects. The bioavailability of this drug depends on food administration and pH value: the water solubility of sorafenib decreases with increasing pH [19]. Furthermore, SFT exhibits higher bioavailability and lower side effects in comparison with sorafenib [20]. SFT showed potentiality like radiosensitizer in the treatment of glioblastoma opposed to sorafenib which not improves the patient survival. Clinical studies demonstrated that these two different behaviors are probably due to the different ability to bind albumin. SFT does not bind albumin, thereby improving its effectiveness compared to sorafenib [21]. All of this makes SFT a more interesting antitumor agent compared to sorafenib.

Recently, the core/shell nanostructures, consisting of a polymeric core and a silica shell, have been considered promising thermal-responsivity drug carriers, thanks to their unique optical responsitivity, and a chemically modifiable surface. The surface of silica is covered by silanol groups, which can react with various chemical agents to conjugate with several kinds of biomolecules and specific ligands [22,23]. Thus, the development of novel hybrid systems, arising from combinations of materials different in nature [24,25] and characterized by unique biological properties, represents a tremendously interesting field in biomedical research [26,27,28].

Several carriers have been developed with the aim of delivering therapeutic agents to specific targeted sites in a controlled manner [29]. A reduction in the intake frequency, and a more uniform effect of the drug, together with an attenuation of drug side-effects, are obtained by employing nano delivery systems [30]. Unfortunately, the high cost of these systems has hindered their widespread dissemination [31,32].

Externally triggerable drug delivery systems show the ability to enhance therapeutic efficacy, while reducing side effects [33,34]. In particular, the unique optical, physical and magnetic properties of inorganic NPs are exploited to reach a selective drug release induced by an external stimulus, such as the application of a magnetic field or light irradiation [35,36]. Light is a versatile and easily tuned external stimulus that can provide spatio-temporal control.

Based on the design of light-responsive drug carriers, different mechanisms of drug activation are involved. Under light irradiation, plasmonic NPs (Ag, Au) convert some part of energy into heat, which is mainly related to the absorption process [37]. Heating efficiency depends on the nature of plasmonic NPs’ optical properties and arises from the resonant interaction of the electromagnetic field at certain frequencies with the oscillations of electrons (plasmons) [38]. These plasmons provide considerable enhancement to an electric field near the NPs’ surface as well as an increase in optical absorption at the resonant frequency [39]. Among the adopted light sources, ultraviolet radiation has been commonly used to induce drug release in hyperbranched polyglycerol micelles [40,41]. Primary limitations of UV-stimulated release are poor tissue penetration (1–2 mm) and its damaging effects on healthy tissues [42]. To avoid them, scientists are shifting the focus towards developing near-infrared (NIR) or visible light-responsive material [43]. However, in principle and taking into account the novelty from fiber-fabrication technology, an injector, including an optical fiber, could be engineered to administer and deliver the UV light radiation to the target side. Technological advances may suggest the use of more sophisticated wirelessly controlled nanowires responding to an electromagnetic field generated by a separate device [44]. This engineering system eliminates the tubes and wires required by other implantable devices that can lead to infections and other complications, whilst activating drug release in the proximity of areas of the body that are often difficult to access.

Furthermore, to overcome the multidrug resistance effect, which is responsible for the low effectiveness of chemotherapeutic agents, light-responsive NP/nanofiber copolymers are employed as drug nanocarriers [45]. In particular, poly(methacrylic acid) (PMA) containing silver (Ag) NPs is a promising light-responsive drug carrier [46], for its good solubility in water and ability to act as good capping agent. As reported in a previous paper [47], upon visible light stimulation, electrospun Ag-PMA nanofibrous scaffold is able to release a significant amount of SFT at a fast rate, thanks to the Ag-mediated laser irradiation heating effect. Further, upon heat stimulation, Ag-PMA nanocolloids provide greater cumulative drug release with respect to the electrospun scaffold. Nowadays, an interesting approach to obtaining polymer capsules is based on the solid core/mesoporous shell (SC/MS) template strategy [48,49], which consists of the infiltration and cross-linkage of polymer components inside the silica porous template shell [50].

In this work, we report the loading of SFT inside Ag-PMA nanocapsules, which were obtained via SC/MS template strategy. The SiO${}_{2}$ template system helped to obtain a well-defined morphology; i.e., hollow capsule-like. The drug was loaded into the silica microparticles (SiO${}_{2}$ template), and followed by the formation of the Ag-PMA polymeric network. Then, after depositing the polymer coating, the SiO${}_{2}$ was dissolved in HF and only the drug-loaded Ag-PMA nanocapsules could in principle be injected into the cell/animal model. Thus, the SiO${}_{2}$ capsules are just useful to improve both drug loading (DL%) and encapsulation efficiency (EE%), compared with previous results without the SC/MS template strategy [47]. The drug release mechanism is mainly induced by laser irradiation treatment wherein the light is specifically tuned to the Ag NPs’ surface plasmon resonance (SPR). The experimental data showed that the irradiated system was able to release a larger amount of drug compared to the non-irradiated system in the same experimental conditions.

This method allows one to store a large amount of a given drug for a long time, and to reach a controlled drug release at the targeted location in the desired time.

A combination of analytical techniques, including dynamic light scattering (DLS), thermogravimetric analysis (TGA), Raman and infrared (FTIR) spectroscopies and scanning and transmission electron microscopies (SEM-EDX/STEM), have been used to investigate the structural and morphological properties of the synthesized carrier. The drug loading and the encapsulation efficiency were evaluated by UV–Vis absorption spectroscopy. Plasmon–enhanced controlled drug release was investigated at different irradiation wavelengths (632 and 420 nm), and upon irradiation close to the resonant wavelength, at two different laser power values of 20 mW cm${}^{-2}$ and 80 mW cm${}^{-2}$, respectively.

## 2. Results and Discussion

### 2.1. SiO${}_{2}$ Template Microparticles

**Figure 1:**
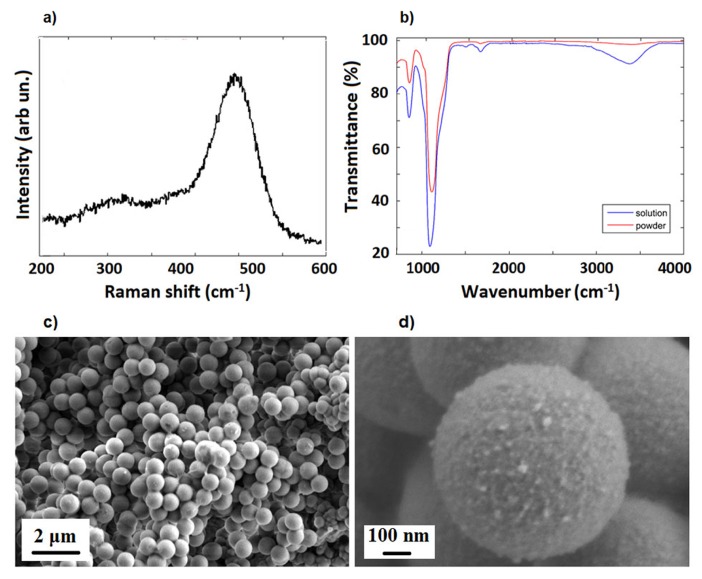
Raman (**a**), FTIR (**b**) and SEM images (**c**,**d**) of SiO${}_{2}$ template microparticles.

SiO${}_{2}$ template microparticles were characterized by FTIR and Raman spectroscopies (see Supplementary Information for experimental details). The Raman broad band centered at 480 cm${}^{-1}$ (see Figure 1a) is associated with Si transverse-optic (TO) vibrational mode, while those smeared at about 300 cm${}^{-1}$ are ascribed to the antisymmetrical and LO stretching modes of Si–O and Si, respectively [51]. On the other hand, FTIR spectra of SiO${}_{2}$ microparticles (powder and solution) show the two typical symmetrical vibration bands of amorphous silica materials. In detail, the bands around 1100 cm${}^{-1}$ and 795 cm${}^{-1}$ are due to Si–O–Si and Si–O stretching and bending vibrational modes, respectively [52]. Moreover, the O–H stretching modes in H-bonded water [53] between 3100 and 3600 cm${}^{-1}$ and the scissor bending vibration modes of water molecules at 1665 cm${}^{-1}$ are observable in the FTIR spectrum of SiO${}_{2}$ microparticles (see Figure 1b). Figure 1c,d report SEM images of the SiO${}_{2}$ particles, showing a good spherical morphology with a diameter of about 1 μm.

### 2.2. SFT-Ag-PMA SiO${}_{2}$ and SFT-Ag-PMA Capsules

**Figure 2:**
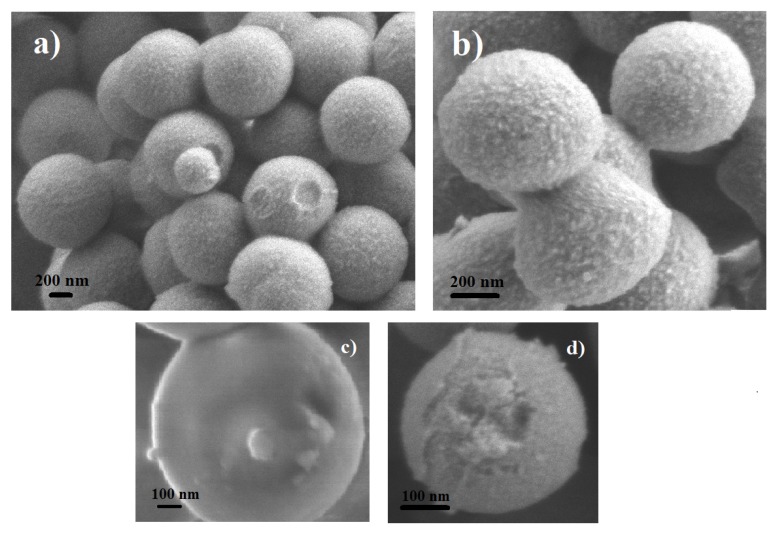
SEM images of SFT-Ag-PMA SiO${}_{2}$ capsules showing a porous structure (**a**,**b**), a smooth surface (**c**) and a layered composition (**d**).

The spherical SFT-Ag-PMA SiO${}_{2}$ capsules are characterized by a well-defined SC/MS porous structure as shown in Figure 2. The capsules are mainly spherical in shape and with an average diameter of about 1 μm. A porous structure characterizes the sample with pores diameter in the 5–25 nm range (Figure 2a,b), as already reported for mesoporous materials [54]. Some SFT-Ag-PMA SiO${}_{2}$ capsules show smooth surfaces with some protuberances (Figure 2c), while others are flaked, showing a layered composition (Figure 2d).

**Figure 3:**
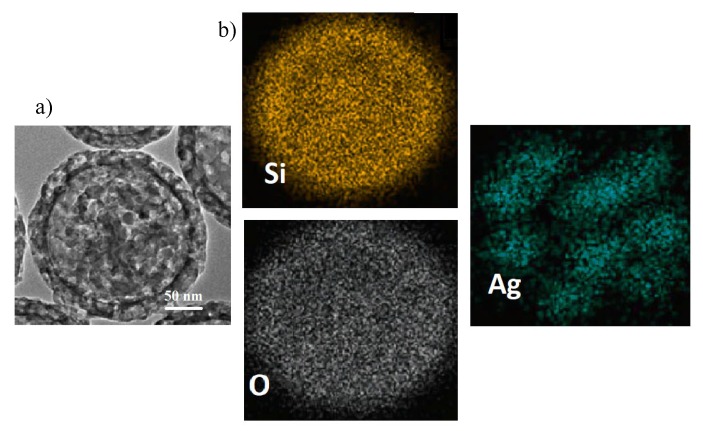
SEM image (**a**) and EDX elemental mapping (**b**) of Si, O and Ag, carried out on a portion accidentally detached from SFT-Ag-PMA SiO${}_{2}$ capsules.

In order to analyze the chemical composition of the inner part of the SFT-Ag-PMA SiO${}_{2}$ capsules, EDX analyses were performed on a portion of the sample that had accidentally detached from the capsule (see Figure 3a). The portion of the cavity structure (inner structure) shows a uniform diameter of about 200 nm, an average pore size of 5 nm and a shell thickness of about 20 nm. This is an effect determined by the high number of protons in the aqueous phase and by the droplet surface. Protons catalyzed the hydrolysis and condensed polymerization of more tetraethyl orthosilicate (TEOS) molecules to generate primary SiO${}_{2}$ nuclei, while the protons on the droplet surface directly captured more primary nuclei of SiO${}_{2}$ and TEOS monomers to form the real microcapsule with thicker shell [55]. On the other hand, EDX mapping analyses show that silicon and oxygen are uniformly distributed in the SFT-Ag-PMA SiO${}_{2}$ capsules, as expected. In particular, Ag NPs seem distributed as “egg yolks” next to each other on the overall area (for the index of the interconnected Ag-PMA network of the capsules see Figure 3b).

**Figure 4:**
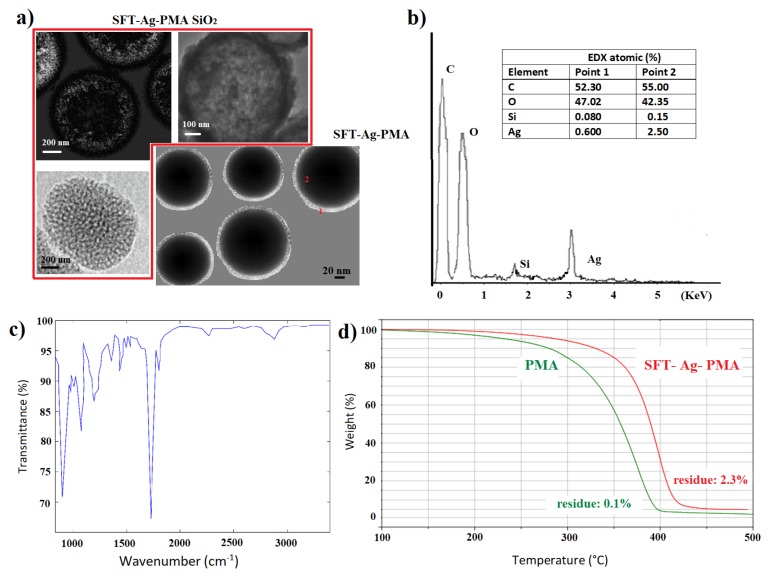
TEM images of SFT-Ag-PMA SiO${}_{2}$ and SFT-Ag-PMA capsules (**a**); EDX and FTIR spectra of SFT-Ag-PMA capsules (**b**,**c**). TGA profiles of PMA (green) and SFT-Ag-PMA (red) capsules (**d**).

The removing of SC/MS silica template induces the formation of hollow core/mesoporous shell (SFT-Ag-PMA) polymer capsules [56,57] (see supplementary materials for experimental details). Morphology and porosity details about SFT-Ag-PMA capsules compared to SFT-Ag-PMA SiO${}_{2}$ capsules were obtained by TEM analysis (see Figure 4a). Capsules show a diameter which varies between 200 and 750 nm, approximately 80% and 25% smaller than the SiO${}_{2}$ template used (Figure 4a), with an evident degree of porosity in both the cores and the shells of the capsules. We outline that the presence of Ag NPs explains why many capsules are “nearly-uniform black” (bottom right image); the size reduction is due to the mechanical force to which the capsules are subjected. Moreover, we cannot exclude that drug incorporation led to a size decrease for the progressive neutralization of the PMA segments in the cross-linked core due to drug binding [58,59]. Nevertheless, the good stability of the sample is indicated by the zeta potential value of −45.57 mV. Furthermore, EDX mapping analyses give indications about the compositions of the capsules. We found: Ag 2.5%, C 55%, O 42.35% and Si 0.15% inside the capsules (Figure 4b), while on the edge of the capsules, as expected, the percentages of Ag and Si were 0.6 and 0.08, respectively. The typical chemical structure of PMA can be easily envisaged in the FTIR absorption spectrum of SFT-Ag-PMA capsules (Figure 4c). Indeed, the band between 2967 and 2683 cm${}^{-1}$ is related to the C-H${}_{x}$ vibrational stretching modes, while the one at 1727 cm${}^{-1}$ is related to the C=O stretching ones. In addition, below 1500 cm${}^{-1}$, the assignments are the following: (i) the bands from 1456 cm${}^{-1}$ to 1370 cm${}^{-1}$—CH${}_{3}$ bending and C-O stretching modes; (ii) the band centered at 1040 cm${}^{-1}$, C–O stretching modes; (iii) the wide one from 1200 to 900 cm${}^{-1}$, C–H${}_{x}$ bending modes [47]. Ultimately, the thermal stabilities of both PMA and SFT-Ag-PMA capsules after SiO${}_{2}$ removal wre evaluated by TGA. The TG curves (Figure 4d) show a higher thermal stability of the SFT-Ag-PMA system compared to PMA capsules. Not relevant residual mass was detected for PMA capsules (∼0.1%). On the contrary, a percentage of residual mass of about 2.3% was estimated in SFT-Ag-PMA capsules, which corresponds to the amount of Ag NPs loaded into the composite.

### 2.3. Drug Release

The SC/MS template strategy allows for improving both drug loading (DL%) ∼13% and encapsulation efficiency (EE%) ∼76% of SFT in Ag-PMA capsules (SFT-Ag-PMA) compared with previous results without an SC/MS template (DL% ∼5.5% and EE% ∼60%) (see [47] and Supplementary Materials for the experimental details).

**Figure 5:**
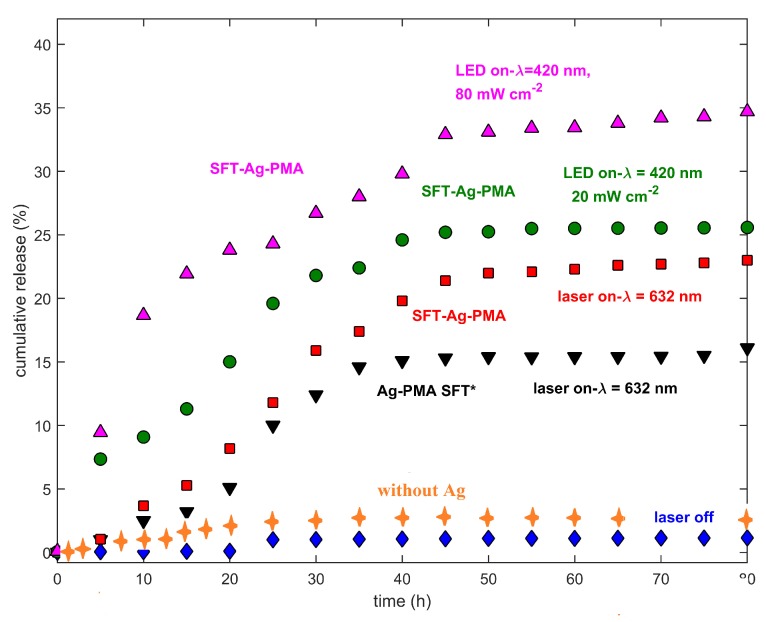
Drug release profiles of SFT-Ag-PMA capsules (red, green and violet symbols), collected at two different laser wavelengths (632 and 420 nm) and power levels (20 and 80 mW cm${}^{-2}$) are reported. Drug release profiles of SFT-PMA capsules (orange crosses), the Ag-PMA SFT* colloid system (black down triangles) and SFT-Ag-PMA capsules without laser irradiation (blue diamonds) respectively, are shown. The cumulative drug released percentages as a function of time are plotted after normalization to the laser power.

Here, we mainly compare the release profiles of SFT from two different drug delivery systems made by the same components (PMA, Ag NPs and SFT) but obtained with two different strategies: (i) solvent evaporation method (Ag-PMA SFT* colloid) and (ii) SC/MS template strategy (SFT-Ag-PMA capsules). Figure 5 reports the cumulative drug release as a function of time, stimulated by lasers of different wavelengths and powers. Slow release kinetics occur when the system is irradiated with the 632 nm laser light; in the first 25 h, about 10% of the encapsulated drug is released and a nearly linear trend between the released drug percentage and the released time is observed. However, the overall drug released by the SFT-Ag-PMA capsules (up triangles, circles and squares) obtained by using the silica template is higher compared with that evaluated for Ag-PMA colloid systems (down triangles). Therefore, SFT-Ag-PMA capsules are a more interesting drug delivery system that can be activated by an external light stimulus, allowing a spatial-temporal and dosage-controlled “on demand” drug release. SFT-Ag-PMA capsules are able to release a higher amount of drug compared to Ag-PMA SFT* colloid system upon the same light irradiation; this is due to higher DL% and EE% values obtained with the new formulation strategy based on silica template.

The release profiles show strong dependencies on laser wavelength and metallic NPs’ SPR [60]. The drug release profiles of SFT-Ag-PMA capsules, irradiated at different wavelengths and laser powers, show that in the early stage (before 25 h), about 25% of the encapsulated drug is released, reaching 35% of release within 80 h, as shown in Figure 5. Certainly, the release mechanism is aided by the Ag-mediated laser irradiation heating effect, making it more effective than the diffusion process alone, so that a higher percentage of SFT is released upon irradiation within 10 h with respect to the non-irradiated sample or the sample irradiated with a source whose excitation wavelength is far from Ag SPR. Finally, we observed that the drug release profiles are characterized by a limited/rapid initial release from the loosely bound surface (times lower than 15 h), followed by two additional phases, corresponding to diffusion and erosion release, as the heating effect induced by laser irradiation increases, with the highest drug released percentages at around 25 h and 40 h [61].

This behavior can be explained taking into account that the rate of drug release is greatly affected by the surface chemistry and physico-chemical properties, such as particle size and shape of the carrier, and the densities of the drug binding sites, since these affect the degradation process. Ag-PMA capsules undergo the drug encapsulation mechanism by altering the pore size of encapsulate, the effective volume, crosslinks, conformation and bubble formation through physico-chemical stimuli (e.g., light and heat). The change in effective volume of the carrier, through the use of the light stimuli, controls the opening mechanism of the Ag-PMA carrier, allowing for controlled drug delivery to take place. In addition, the reduction of Ag-PMA crosslinks, used for drug encapsulation and stabilizing the inner part of NPs, rules the change in effective volume and internal permeability of the carrier. Furthermore, light causes a reversible change in the linker configuration which results in a decrease of the encapsulation volume, and mainly, a faster drug release rate [62]. Hence, the light-heating mechanism dominates the normal diffusion one when the nanocarrier is irradiated with a light source whose wavelength is close to the Ag NPs’ SPR. This, in turn, determines the most effective heating of the temperature responsive SFT-Ag-PMA capsules and their significant drug release enhancement. Even if it is evident that all these parameters affect the rate and efficiency of drug release, further studies are necessary to optimize the physico-chemical properties of the composite in order to effectively control “on demand” the release of the drug at the desired target.

**Figure 6:**
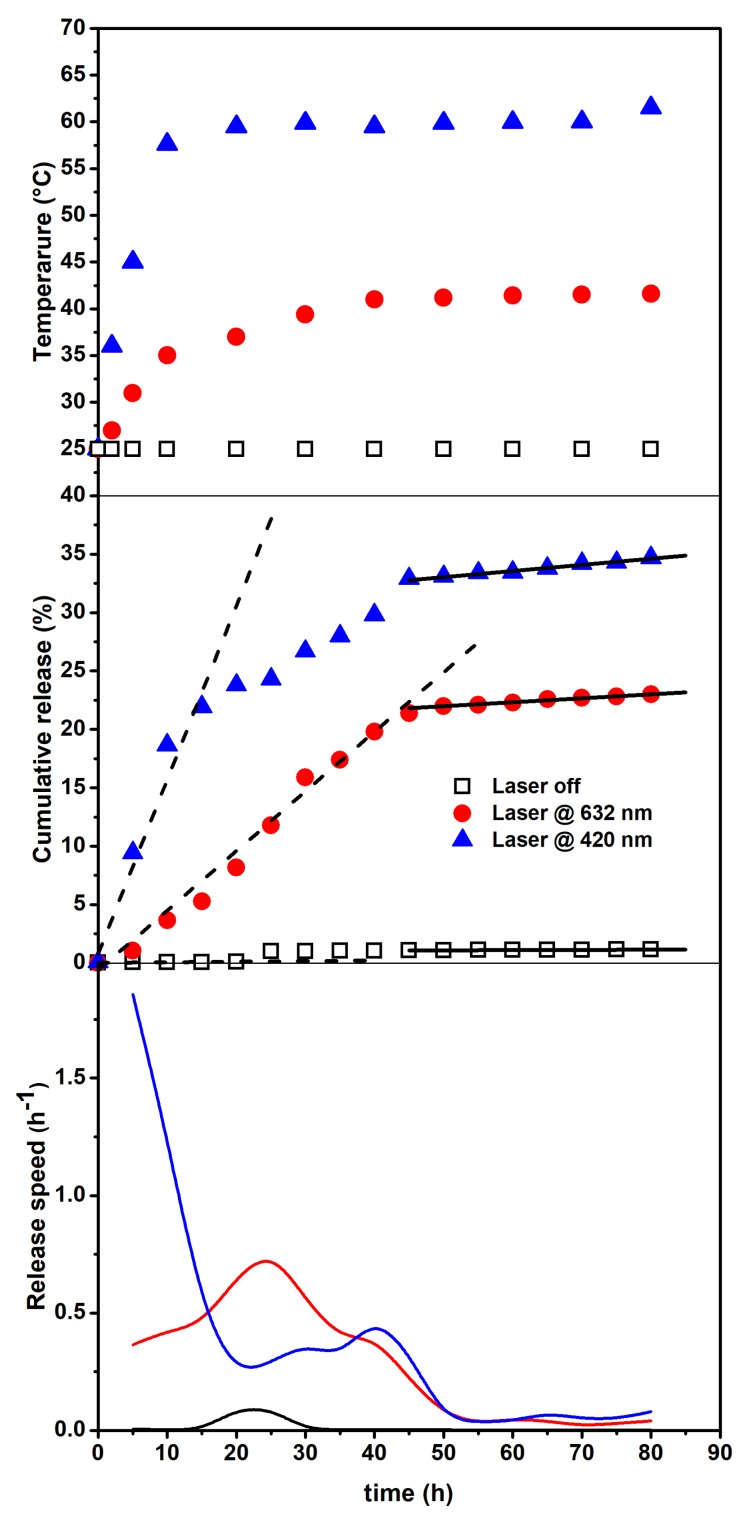
Temperature changes vs. time (upper panel). Cumulative release (central panel) from the SFT-Ag-PMA capsules under the 632 (circles) and 420 nm (triangles) light and with no light exposure (squares). Dashed and solid lines represent linear fits of the data in limited time intervals (see text). Release speed (bottom panel) obtained by the derivatives of the experimental data.

To characterize the observed experimental behavior over time, we compared the cumulative drug release (central panel) from the same set of SFT-Ag-PMA capsules exposed (or not) to different light stimuli (see Figure 6).

The upper panel shows the temperature change vs. time, indicating that a primary release (due to the photothermal driven force) followed by a secondary release (resulted from the temperature- independent polymer degradation) takes place. Ultimately, the bottom panel reports the derivatives of the experimental data, evidencing the different behavior in the release speed and also indicating when the major changes occur. The behavior of the cumulative drug release vs. time, also reflected by the corresponding derivative and the temperature changes, suggests that the release occurs at least in two steps related to: (i) a thermally activated drug diffusion mechanism and, (ii) at longer times, a much slower polymer swelling/degradation one [63]. The release speed behavior indicates that, for the irradiated system, major changes take place within 50 h, with a significantly higher efficiency for the irradiation on resonance (420 nm). Therefore, by assuming that the first step of release can be treated as a zero-order kinetics (mainly associated to polymer matrix opening effects), we have evaluated the rate of release by a linear fit in the corresponding time intervals. The obtained results are reported in the central panel of Figure 6 as dashed lines and show a value as high as 1.5% h${}^{-1}$ for the irradiation on resonance case (420 nm), while release is almost negligible for the non-irradiated sample. Next, the photo-thermally activated drug diffusion mechanism slows down (reduced slope)—and at longer times (solid lines), although the overall efficiencies are very different, the release rates are very small and nearly similar—so that can be associated to mostly temperature-independent polymer erosion processes [64,65,66].

For an attentive reader, it immediately emerges that the temperatures reached by irradiating on resonance (420 nm close to Ag SPR) are quite high. For a typical tumor treatment, during thermal steady state, temperature generally varies from 37 to 43 °C, since temperatures higher than 45 °C can cause harmful effects on healthy tissues. However, high temperatures are particularly useful if there is a focus of causing necrosis in a tumor [67]. We outline that, although release times are high with respect to other delivery systems, our composites have been developed to achieve high encapsulation and retention of drugs, while maintaining prolonged circulation after their administration. This will result in the accumulation of the drug at the target site to a greater extent than in healthy tissues, and will, in principle, lead to improved therapeutic outcomes.

## 3. Materials and Methods

### 3.1. Materials and Summary of Used Samples

Powder of silver nitrate (AgNO${}_{3}$), poly(methacrylic acid) sodium salt ([CH${}_{2}$C(CH${}_{3}$)(CO${}_{2}$Na)]${}_{n}$, Mw = 9500), (TEOS), *N*-(3-Dimethylaminopropyl)-*N*′-ethylcarbodiimide hydrochloride (EDC), n-octadecyltrimethoxysilane (ODTMS), 1,4-Dithiothreitol (DTT), 5,5′-Dithiobis(2-nitrobenzoic acid) (DTNB or Ellman’s reagent), poly(vinylpyrrolidone) (PVPON) Mw = 55 kDa, and chloramine T trihydrate (CaT) were purchased from Sigma-Aldrich. Pyridine dithioethylamine hydrochloride (PDA-HCl) was purchased by Alfa chemistry. Acros Organics supplied polyvinyl alcohol (PVA, Mw = 86,000). Faculty of Pharmacy of Messina University kindly provided sorafenib Tosylate (SFT). Ag content in Ag-PMA was determined by GF-AAS using a Varian 220/Zeeman atomic absorption spectrometer (AAS Mulgrade, Victoria, Australia), equipped with a single-element hollow cathode lamp and a Varian PSD autosampler. For the analysis, samples were diluted 1:5000 (*v*:*v*) with 0.2% HNO${}_{3}$ (AAS grade). The analytical method was validated according to the ICH guidelines (International Council for Harmonisation of Technical Requirements for Pharmaceuticals for Human Use, 2005). The linearity was >0.999, the precision (expressed as relative standard deviation) 0.552 and the detection limit 5.0 ng/mL.

A list of the investigated samples and some formulation details are reported in Table 1 while the extended synthesis protocols are shown in Supplementary Materials.

### 3.2. Drug Release

Drug release was assessed using the dialysis membrane (DM) method. The dialysis bag (MWCO = 3.5–5 kDa Spectra/Porr) was filled with 4 mg of the Ag-PMA loaded with SFT in a SiO${}_{2}$ template. After SiO${}_{2}$ removal, the sample was dipped in PBS (external bag: 15 mL) and kept at 37 °C under constant stirring. At fixed intervals, 1 mL of the release medium was withdrawn, replaced with an equal volume of fresh PBS and analyzed by UV–Vis optical spectroscopy. All the experiments were carried out in duplicates. For the release experiments stimulated by light irradiation, a CW He–Ne (λ = 632.8 nm; energy density P = 21 mW cm${}^{-2}$) and an LED source (λ = 420 nm; energy density P = 30 mW cm${}^{-2}$) were used. Then, the cumulative released drug optical absorbance values are plotted as a function of time after normalization to the laser power.

## 4. Conclusions

SFT-Ag-PMA capsules obtained by using sacrificial silica microparticle templates, showed better DL and EE percentages compared to Ag-PMA SFT* colloid solution (13 vs. 5.5 DL%, 76 vs. 60 EE%). Drug release was driven by external light stimuli, by tuning the light irradiation wavelength and power intensity. The major drug encapsulation was matched by a better release capacity of SFT-Ag-PMA capsules (∼35%), with respect to the non-irradiated sample (∼2%) and the Ag-PMA SFT* colloid system (∼13%). Despite the interesting results concerning drug retention and its prolonged circulation after administration, several studies must be focused on improving the amount of drug released as a function of time. For this purpose, a further systematic study varying morphological and chemical compositions of these composites should be carried out, simultaneously testing the therapeutic efficiency of SFT-Ag-PMA capsule system.

## Figures and Tables

**Table 1:** Formulation details for the corresponding samples. See the supplementary materials for more details.

Sample	Formulation Details
SFT-Ag-PMA capsules	silver polymer capsules loaded with the drug, sorafenib tosylate (SFT) prepared by using solid core/mesoporous shell (SC/MS) template strategy
SFT-PMA capsules	polymer capsules loaded with the drug, sorafenib tosylate (SFT) prepared by using solid core/mesoporous shell (SC/MS) template strategy
Ag-PMA capsules	silver polymer capsules prepared by using solid core/mesoporous shell (SC/MS) template strategy
PMA capsules	polymer capsules prepared by using solid core/mesoporous shell (SC/MS) template strategy
Ag-PMA colloid solution	colloidal solution of silver polymer NPs prepared by a formulation strategy reported in Ref. [47]. Afterwards Ag-PMA NPs are loaded with SFT (Ag-PMA SFT*)

## Supplementary Materials

The following are available online: Experimental procedure to prepare: (i) SFT-Ag-PMA capsules, (ii) SFT-PMA capsules, (iii) Ag-PMA capsules, (iv) PMA capsules and (v) Ag-PMA SFT* colloid solution. Evaluation of drug loading % and encapsulation efficiency % for both SFT-Ag-PMA capsules and Ag-PMA SFT* colloid solution. Characterization techniques to reach Ag-PMA SFT* colloid solution. Evaluation of stability and size distribution of Ag-PMA colloid solution. Figure S1: UV-vis absorbance spectra; STEM images of Ag-PMA solutions and Ag NP size distribution histogram.

## Abbreviations

The following abbreviations are used in this manuscript:

SPR	Surface Plasmon Resonance
NPs	NanoParticles
DL	Drug Loading
PMA	poly-methacrylic acid, sodium salt
EE	Encapsulation Efficiency
MSPs	Mesoporous Silica Particles
PS	Polystyrene
Ag	Silver
SC/MS	Solid Core /Mesoporous Shell
DLS	Dynamic Light Scattering
TGA	Thermogravimetric Analysis
FTIR	Infrared
SEM-EDX/STEM	Scanning/Transmission Electron Microscopes
SFT	Sorafenib-Tosylate
TEOS	Tetraethyl Orthosilicate
