# Supplementary material for: Plasmon-Enhanced Controlled Drug Release from Ag-PMA Capsules
Source: Molecules. 2020 May 11;25(9):2267. doi: 10.3390/molecules25092267 (PMC7248805; doi:10.3390/molecules25092267)
Supplement: Supplementary file 1 [file molecules-25-02267-s001.pdf]

Supplementary Materials: Plasmon- enhanced controlled drug release from Ag-PMA capsules

Giulia Neri *, Carmelo Corsaro and Enza Fazio

1. Experimental Details

1.1. Experimental procedure to prepare SFT-Ag-PMA capsules

Here we report the detailed experimental procedure used to prepare SFT-Ag-PMA capsules.

Preparation of SiO_2 template microparticles. The synthetic procedure involves two steps [1]. In the first step, solid core silica particles of ca. 200 nm were obtained, afterwards an external mesoporose shell of ca. 20 nm was formed onto the silica solid core, to reach the solid core/mesoporous shell (SC/MS) particles. Ethanol (1 mL), deionized (Milli-Q) water (135 µL) and ammonia 28% solution (113 µL) were mixed and heated up to 25 °C. Then, tetraethoxysilane (TEOS, 75 µL) was added and the solution was stirred 1 h at 25 °C, thus the silica solid core was obtained. In the meantime, TEOS (65 µL) and ODTMS (13 µL) were mixed and subsequently dropwise added into the solid core solution under stirring at 25 °C. Then, the solution was stirred 35 min at 25 °C. The particles were dried in oven at 70 °C, followed by an incubation at 550 °C in a furnace under air flow. Thus, SiO_2 template microparticles were obtained as powder.

Sorafenib Tosylate loading in SiO_2 microparticles. The active pharmaceutical ingredient in the marked oral tablet consists of 274 mg of Sorafenib tosylate. Nexavar: European Public Assessment Reports (EPAR)-Scientific Discussion. European Medicines Agency (EMA).

SFT was loaded in SiO_2 with slight modifications with respect to the procedure reported in Ref. [2]. 1 mg of hydrophobic drug was dissolved in 10 mL of dichloromethane, then 5 mg of SiO_2 microparticles were added. The system was stirred for 6 h at r.t. To remove the unloaded drug, the mixture was centrifugated at 3000 rpm for 2 min, the precipitate was collected, washed twice and dried in a vacuum desiccator to remove the dichloromethane. The amount of drug in the supernatant was evaluated by UV absorption spectroscopy following the absorbance signal of SFT at the 265 nm. Then 13% drug loading (DL%) and 76% encapsulation efficiency (EE%) values in SiO_2 microparticles were computed by using the following equations [3]:

$$\boldsymbol{DL\%} = \frac{100 \times (\text{Weight of Drug Encapsulated})}{(\text{Weight of Drug Encapsulated + Weight of SiO}_2\text{ particles})}$$

$$\boldsymbol{EE\%} = \frac{100 \times (\text{Weight of Drug Encapsulated in SiO}_2\text{ particles})}{(\text{Weight of Drug Used in Encapsulation Method})}$$

Preparation of Ag-PMA colloid solution. To obtain Ag-PMA colloid solution, a two-step photoinduced reduction strategy under UV irradiation was just developed by some of us [4]. Briefly, a PMA 30 wt% (in Milli-Q water) solution was prepared and $AgNO_3$ powder was added in 10:1 $AgNO_3$/PMA molar ratio. The mixture was then stirred 5 h at r.t. In order to optically reduce the silver ions, the mixture was irradiated at a UV light density of 470 nW/cm^2. Afterwards, the mixture was also irradiated with a UV light radiation density of 378 $\mu W/cm^2$ for 5 hours. The system was stable up to one month.

Synthesis of Ag-PMASH. The synthesis was performed as reported in literature [5]. Ag-PMA solution (190 mg of 30 wt% solution) was prepared and diluted in 3 mL of PB solution (pH 7.4, 50

mM). Then, EDC (43 mg, 0.22 mmol) was added to the solution and the system was left under stirring for 30 min at r.t. Afterward, PDA-HCl (29 mg, 0.13 mmol) was added and the mixture was stirred for 24 h at r.t. In order to purify Ag-PMA-PDA compound, the mixture was dialyzed against Milli-Q water for 3 days and then lyophilized to give Ag-PMA-PDA like a powder. To obtain free thiol groups, the 2-mercaptopyridine moieties must be cleaved from Ag-PMA-PDA. In detail, Ag-PMA-PDA was solubilized in 0.5 M DTT solution in MOPS buffer (20 mM, pH 8.0) to reach a final concentration of 100 g/L and the solution was stirred 30 min at 37 °C. Then, the obtained Ag-PMASH was diluted up to 100 g/L by using NaOAc buffer solution (pH 4.0, 50 mM).

Determination of thiol group content. The thiol content was spectrophotometrically evaluated by using Ellman's reagent. In details, two different solutions, 50 mM DTNB (Ellman's reagent) in sodium acetate and 1 M TRIS solution at pH 8.0 were prepared. In the meantime, SH standard calibration curve by using acetyl Cysteine, staring from 10 mM concentration, was obtained. 10 μL of sample solution were added into 990 μL of DTNB solution and the mixture was incubated for 5 min at r.t. Finally, the optical absorbance at 412 nm was measured. 350±4.5 μg/mL SH content was estimated.

Preparation of SFT-Ag-PMA capsules. SFT loaded-SiO_2 microparticles were dispersed in 50 μL of NaOAc buffer solution, by vortex and sonication treatments. 5 μL of PVPON solution 4 g/L in 50 mM NaOAc buffer was added into SFT loaded- SiO_2 particles suspension and incubated overnight. Then, the PVPON-SiO_2 system was washed and resuspended in NaOAc buffer, by using the same method previously reported for SiO_2 microparticles. Finally, PVPON-SiO_2 system was resuspended in 50 μL of NaOAc buffer solution. At the same time, 50 μL of Ag-PMASH suspension (4 g/L in 50 mM NaOAc buffer) were added into PVPON-SiO_2 suspension and incubated overnight. The obtained system was washed and resuspended in NaOAc buffer three times and finally in 50 μL of NaOAc buffer solution. Then, the interconnection of infiltrated polymer was allowed by cross-linkage processes via thiol oxidation by using CaT 2mM solution in 10mM MES buffer, the system was stirred 15 min. To release PVPON, the capsule were repeatedly washed with PB solution (10 mM, pH 7.2) [6]. Thus SFT-Ag-PMA SiO_2 capsules were obtained. In order to form the SFT-Ag-PMA capsules the silica template was removed by using HF 5 mM solution for 5 min, followed by three centrifugation/washing cycles in PB solution at 2000 rpm for 12 min [7].

1.2. Experimental procedures used to prepare SFT-PMA capsules

Preparation of SFT-PMA capsules. These capsules were prepared by following the same method adopted for SFT-Ag-PMA capsules. In this case, PMA was not decorated with Ag NPs.

1.3. Experimental procedures used to prepare Ag-PMA capsules

Preparation of Ag-PMA capsules. These capsules were prepared by following the same method adopted for SFT-Ag-PMA capsules, but without the drug loading step.

1.4. Experimental procedures used to prepare PMA capsules

Preparation of PMA capsules. These capsules were prepared by following the same method adopted for Ag-PMA capsules. In this case, PMA was not decorated with Ag NPs.

1.5. Experimental procedures used to prepare Ag-PMA SFT colloid solution*

Here we briefly report the Ag-PMA SFT* colloid formulation strategy [4].

Preparation of Ag-PMA colloid solution. To obtain Ag-PMA colloid solution, we performed a two-step photoinduced reduction strategy under UV irradiation. Briefly, a PMA 30 wt% (in Milli-Q water) solution was prepared and $AgNO_3$ powder was added in 10:1 $AgNO_3$/PMA molar ratio. The mixture was stirred for 5 h at r.t. In order to optically reduce the silver ions, the mixture was irradiated

at a UV light density of 470 nW/cm^2. Afterwards, the mixture was also irradiated with a UV light radiation with density of 378 μW/cm^2 for 5 hours. The system was stable up to one month.

Sorafenib Tosylate (SFT) loading in Ag-PMA colloid solution (Ag-PMA SFT*). SFT was solubilized in methanol to reach a solution at the final concentration of 200 ppm. 0.5 g of Ag-PMA colloid solution was added into 20 mL of SFT solution, following by ultrasonication treatment (20 min, Sonics VCX 130). The mixture was stirred for 24 h at r.t. Then, the concentration of SFT in the multicomponent colloidal solution was estimated following spectrophotometrically the drug absorbance signal at 265 nm. A drug loading of 5.5% and an encapsulation efficiency of about 60% were inferred.

$$\boldsymbol{DL\%} = \frac{100 \times \text{(Weight of Drug Encapsulated)}}{\text{(Weight of Drug Encapsulated + Weight of polymer matrix)}}$$

$$\boldsymbol{EE\%} = \frac{100 \times \text{(Weight of Drug Encapsulated in polymer matrix)}}{\text{(Weight of Drug Used in Encapsulation Method)}}$$

1.6. Characterization techniques

A Spectrum 100 Perkin-Elmer spectrometer working in ATR configuration was used to carry out FTIR measurements in the range 900-4000 cm^{-1}. Raman scattering response of the SiO_2 capsules was investigated after the deposition of some drops of the solution on a CaF_2 substrate. Micro-Raman spectra were excited by the 532 nm radiation of a 30 mW diode laser, for an integration time of 60 s. The backscattered radiation, collected by an Olympus BX40 microscope optics using a 100X objective lens, was analyzed by an XploRA 1800 cm^{-1} monochromator equipped with a Peltier CCD sensor. A Zeiss-Gemini 2 electron microscope, operating at 150 kV, is the apparatus used for SEM analyses. When the measurements were carried out in the transmission mode (STEM), the accelerating voltage was 30 kV. SEM apparatus is coupled with a Quantax EDX spectrometer to carry out energy dispersive X-ray (EDX) analysis. The EDX detected pear-shaped dimension is about 0.7 μm. A Horiba NanoParticle Analyzer SZ-100 was used to evaluate the Zeta potential using a laser Doppler method, based on the principle of electrophoretic mobility under an electric field. The thermal degradation of the samples was studied by thermogravimetric analysis (TGA) under air flow, also to estimate the percentage of Ag loaded into the nanocomposite. A Mettler Toledo TGA 851 apparatus (horizontal balance mechanism) was used. The sample weight is 3 mg. The thermogravimetric weight loss curve was recorded as a function of temperature. The balance sensitivity was 0.5 μg. The weight loss was calculated by the difference between the weights at r.t. and at 450 °C.

2. Stability and size distribution of Ag-PMA colloid solution

In order to investigate the colloidal stability of the Ag-PMA system, the colloids were irradiated with the 6W UV lamp for 1 hour and they were re-exposed to the UV light (radiation density of 378 μW/cm^2) by using a 25W UV lamp for 5 hours. No appreciable change in terms of intensity and lineshape of the Ag surface plasmon resonance (SPR) peak was observed after the second step of irradiation and also after a month from the formulation (Fig S1a). This finding proved that: *i)* during the second UV irradiation step, the polymer reticulate limits any further Ag-NP nucleation, growth and aggregation; *ii)* colloids are fairly stable. Moreover, an average size lower than 10 nm was estimated for the 80% of the Ag NPs, while the diameter of the remaining ones does not exceed 25 nm (Fig. S1b-d) [4].

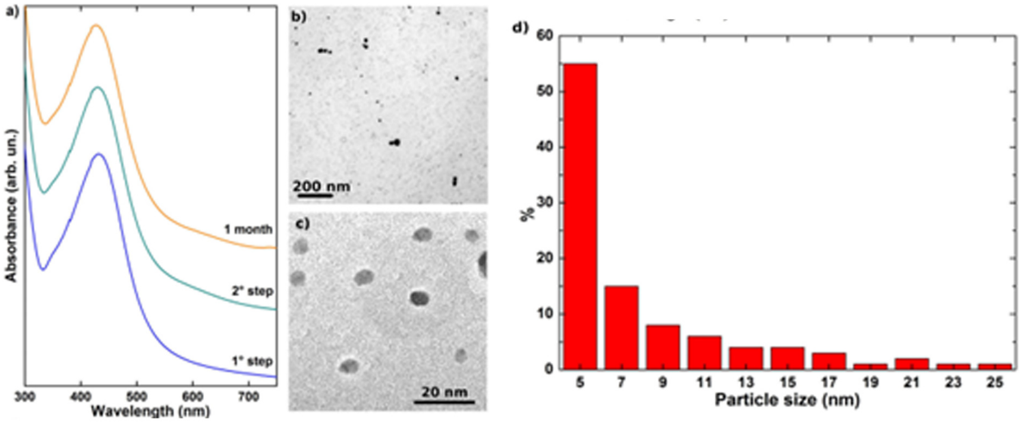

Figure S1. a) UV-vis absorbance spectra; (b, c) STEM images of Ag-PMA solutions after the second irradiation step and (d) Ag NP size distribution histogram. Figure adapted with permission from Neri et al. Current Nanomaterials 4, 32-38 (2019) [4].

Abbreviations

The following abbreviations are used in this Supplemental:

SPR	Surface Plasmon Resonance
NPs	NanoParticles
DL	Drug Loading
PMA	poly-methacrylic acid,5 sodium salt
EE	Encapsulation Efficiency
MSPs	Mesoporous Silica Particles
ODTMS	n-octadecyltrimethoxysilane
PS	Polystyrene
Ag	Silver
PB	phosphate buffer
SC/MS	Solid Core /Mesoporous Shell
DLS	Dynamic Light Scattering
TGA	Thermogravimetric Analysis
FTIR	Infrared
SEM-EDX/STEM	Scanning/Transmission Electron Microscopes
SFT	Sorafenib-Tosylate
TEOS	Tetraethyl orthosilicate
EDC	N-(3-Dimethylaminopropyl)-N′-ethylcarbodiimide hydrochloride
PDA-HCl	pyridine dithioethylamine hydrochloride
DTT	1,4-dithiothreitol
MOPS	(3-(N-morpholino)propanesulfonic acid)
NaOAc	sodium acetate
DTNB or Ellman's reagent	5,5′-dithiobis(2-nitrobenzoic acid)
TRIS	tris(hydroxymethyl)aminomethane
PVPON	poly(vinylpyrrolidone)
CaT	chloramine T trihydrate
MES	2-(N-morpholino)ethanesulfonic acid
HF	fluorhydric acid
UV-vis	ultraviolet–visible
STEM	scanning transmission electron microscopy

References

1. Goethals, E.C.; Shukla, R.; Mistry, V.; Bhargava, S.K.; Bansal, V. Role of the Templating Approach in Influencing the Suitability of Polymeric Nanocapsules for Drug Delivery: LbL vs SC/MS. *Langmuir* **2013**, *29*, 12212–12219, [https://doi.org/10.1021/la4024103]. PMID: 23998648, doi:10.1021/la4024103.
2. Wang, Y.; Yan, Y.; Cui, J.; Hosta-Rigau, L.; Heath, J.K.; Nice, E.C.; Caruso, F. Encapsulation of Water-Insoluble Drugs in Polymer Capsules Prepared Using Mesoporous Silica Templates for Intracellular Drug Delivery. *Advanced Materials* **2010**, *22*, 4293–4297, [https://onlinelibrary.wiley.com/doi/pdf/10.1002/adma.201001497]. doi:10.1002/adma.201001497.
3. Wan, X.; Beaudoin, J.J.; Vinod, N.; Min, Y.; Makita, N.; Bludau, H.; Jordan, R.; Wang, A.; Sokolsky, M.; Kabanov, A.V. Co-delivery of paclitaxel and cisplatin in poly(2-oxazoline) polymeric micelles: Implications for drug loading, release, pharmacokinetics and outcome of ovarian and breast cancer treatments. *Biomaterials* **2019**, *192*, 1 – 14. doi:https://doi.org/10.1016/j.biomaterials.2018.10.032.
4. Neri, G.; Spadaro, S.; Barreca, F.; Santangelo, S.; Neri, F.; Fazio, E. Electrospun Ag/PMA Nanofibrous Scaffold as a Drug Delivery System. *Current Nanomaterials* **2019**, *4*, 32–38. doi:doi:10.2174/2405461504666190416144047.
5. Chen, X.; Cui, J.; Ping, Y.; Suma, T.; Cavalieri, F.; Besford, Q.A.; Chen, G.; Braunger, J.A.; Caruso, F. Probing cell internalisation mechanics with polymer capsules. *Nanoscale* **2016**, *8*, 17096–17101. doi:10.1039/C6NR06657G.
6. Zelikin, A.N.; Li, Q.; Caruso, F. Disulfide-Stabilized Poly(methacrylic acid) Capsules: Formation, Cross-Linking, and Degradation Behavior. *Chemistry of Materials* **2008**, *20*, 2655–2661, [https://doi.org/10.1021/cm703403p]. doi:10.1021/cm703403p.
7. Zelikin, A.N.; Quinn, J.F.; Caruso, F. Disulfide Cross-Linked Polymer Capsules: En Route to Biodeconstructible Systems. *Biomacromolecules* **2006**, *7*, 27–30, [https://doi.org/10.1021/bm050832v]. PMID: 16398494, doi:10.1021/bm050832v.
